# Coloración de Ziehl-Neelsen en el laboratorio de patología: rendimiento y contribución al diagnóstico de micobacterias en el lavado broncoalveolar

**DOI:** 10.7705/biomedica.6347

**Published:** 2022-09-02

**Authors:** Ariel A. Arteta, Luis F. Arias, Claudia E. Cadavid

**Affiliations:** 1 Departamento de Patología, Universidad de Antioquia, Medellín, Colombia Universidad de Antioquia Universidad de Antioquia Medellín Colombia; 2 Grupo de Investigaciones en Patología, Universidad de Antioquia Medellín, Colombia Universidad de Antioquia Universidad de Antioquia Medellín Colombia; 3 Laboratorio Clínico, Área de Microbiología Clínica, Hospital San Vicente Fundación, Medellín, Colombia Hospital San Vicente Fundación Medellín Colombia

**Keywords:** tuberculosis/diagnóstico, lavado broncoalveolar, sensibilidad y especifcidad, Colombia, Tuberculosis/diagnosis, bronchoalveolar lavage, sensitivity and specifcity, Colombia

## Abstract

**Introducción.:**

La coloración de Ziehl-Neelsen, con más de 100 años de uso, continúa vigente mundialmente.

**Objetivo.:**

Comparar el rendimiento de las pruebas diagnósticas utilizadas para la determinación de micobacterias en el laboratorio clínico de patología en muestras de lavado broncoalveolar.

**Materiales y métodos.:**

Se revisaron retrospectivamente 737 muestras de lavado broncoalveolar procesadas en el 2019 y el 2020 en el Hospital San Vicente Fundación (Medellín, Colombia) y se compararon las características de tres pruebas diagnósticas realizadas en paralelo: la reacción en cadena de la polimerasa (PCR) para micobacterias con detección de resistencia, el cultivo, y la coloración de Ziehl-Neelsen.

**Resultados.:**

Se catalogaron como enfermos a 93 de los 737 pacientes a partir de los resultados positivos en alguna de las tres pruebas. El cultivo tuvo una sensibilidad de 0,80, la PCR una de 0,76 y la coloración de Ziehl-Neelsen una de 0,51. Sin embargo, solo 5 de 75 (6,5 %) cultivos fueron positivos a las cuatro semanas y el resto lo fue a las ocho semanas. La PCR combinada con la coloración de Ziehl-Neelsen mejoró la sensibilidad de la PCR por sí sola, de 0,76 a 0,88, diferencia que fue estadísticamente signifcativa (p=0,022)

**Conclusión.:**

En las muestras de lavado broncoalveolar, el cultivo sigue siendo la prueba con mejor sensibilidad. El uso conjunto de la prueba de PCR y la coloración de ZiehlNeelsen mejora signifcativamente la sensibilidad de la primera, lo que compensa la demora relativa en la entrega de los resultados debida al tiempo requerido para la tinción de Ziehl-Neelsen.

## Abstract (en)

**Introduction:**

With more than 100 years of use, the Ziehl-Neelsen stain is still currently used worldwide.

**Objective:**

To compare the performance of diagnostic tests used to determine mycobacteria in the clinic and pathology laboratory in bronchoalveolar lavage samples.

**Materials and methods:**

We retrospectively reviewed 737 bronchoalveolar lavage samples from 2019 to 2020 in the San Vicente Fundación hospital (Medellín, Colombia) comparing the performance of three tests done in parallel: mycobacteria and resistance PCR, culture, and Ziehl-Neelsen stain.

**Results:**

In total, 93/737 patients were classifed as sick due to a positive result in any of the three tests. The culture, PCR, and Ziehl-Neelsen stain had a sensibility of 0.80, 0.76 y 0.51, respectively. However, only 5/75 (6.5%) of the positive cultures had results within the frst four weeks and the rest in eight weeks. The PCR test combined with the Ziehl-Neelsen stain improved the sensibility of the PCR test alone from 0.76 a 0.88, a change that was statistically signifcant (p = 0.022).

**Conclusion:**

At least in bronchoalveolar lavage samples, culture is still the test with better sensibility. The use in parallel of the PCR test and the Ziehl-Neelsen stain improved in a statistically signifcant manner the performance of the PCR test alone, regardless of the higher turnaround time of the Ziehl-Neelsen stain.

La tuberculosis es una enfermedad crónica contagiosa de tipo granulomatoso y potencialmente mortal, causada por bacterias Gram positivas ácido-alcohol resistentes del género *Mycobacterium*, que afecta predominantemente los pulmones ^1^.

La incidencia de la tuberculosis ha cambiado a lo largo de la historia. La enfermedad se presentaba de manera esporádica hasta la primera mitad del siglo XVIII, pero, durante la revolución industrial, el número de casos aumentó, probablemente por la mayor densidad poblacional y las precarias condiciones de vida imperantes ^2^. Su incidencia disminuyó con la introducción de la vacuna en 1921, y el descubrimiento y desarrollo de medicamentos antimicrobianos efectivos contra la infección ^3^.

Con excepción de las pandemias, a nivel mundial, la enfermedad es la novena causa de muerte y la primera debida a un único agente infeccioso, con cerca de 1,2 millones de muertes en el 2019 entre pacientes negativos para HIV y 208.000 en pacientes positivos ^4^.

Colombia es un país cuyas condiciones sociales y económicas favorecen la aparición de la tuberculosis. En el 2018, se reportaron 14.436 casos de todas las formas de la enfermedad, estimándose una incidencia de 26,9 por 100.000 habitantes. La tuberculosis pulmonar representó la mayoría de los casos, con 82,7 % (n=11.940 casos), en tanto que los casos de coinfección con HIV alcanzaron el 9,7 % (n=1.336 casos). La tasa de incidencia de la enfermedad en el país ha tenido una clara tendencia al alza en los últimos seis años, pasando de 23 por 100.000 habitantes en el 2013 a 26,9 por 100.000 en el 2018 ^4^. Actualmente, puede ser inclusive mayor debido al incremento relativo de la población en situación de vulnerabilidad, de la población en condición de pobreza y desnutrición, y del número de migrantes de países vecinos y de habitantes de la calle, entre otras razones.

No obstante, la incidencia de tuberculosis no solo aumenta por las condiciones sociales. En la actualidad, el control inmunológico de la primoinfección está siendo puesto a prueba por situaciones como el incremento del número de pacientes positivos para HIV, el uso de esteroides como parte del tratamiento para la infección por SARS-Cov-2, la multirresistencia de las micobacterias y la administración de medicamentos inmunosupresores en pacientes con trasplantes o para el tratamiento de enfermedades reumatológicas y neoplásicas ^5^^-^^8^. Por ello, se necesitan aproximaciones diagnósticas que nos permitan la demostración rápida y precisa de la micobacteria mediante técnicas de laboratorio complementarias.

En Colombia, la definición operativa del caso de tuberculosis está contemplada en la Resolución 227 de 2020 del Ministerio de Salud y Protección Social, en la cual se señalan tres tipos en la categoría de caso confirmado:

1. por laboratorio, es decir, con resultado positivo en alguna de las pruebas de laboratorio (coloración de Ziehl-Neelsen, cultivo o prueba molecular);
2. por clínica, o sea, un caso en el que las pruebas de laboratorio son negativas, pero los hallazgos clínicos, radiológicos e histopatológicos son sugestivos de la enfermedad y el paciente reacciona positivamente a la prueba terapéutica, y
3. por nexo epidemiológico, caso en que, a pesar de las pruebas bacteriológicas negativas, la persona ha tenido contacto estrecho con un paciente con diagnóstico de tuberculosis, y tiene hallazgos clínicos, radiológicos e histopatológicos sugestivos de la enfermedad, además de una mejoría con la prueba terapéutica ^4^.

Una de las coloraciones más utilizadas en el mundo para confirmar la presencia de micobacterias es la coloración de Ziehl-Neelsen (figura 1) ^9^. Desde hace algún tiempo, en el Laboratorio de Patología se utiliza de manera sistemática la coloración de Ziehl-Neelsen en todas las muestras de lavado broncoalveolar, como parte del procedimiento estándar de operación. En muy pocos estudios locales se ha analizado este procedimiento en este tipo de muestras, pues la sensibilidad y especificidad de la PCR son de 100 y 99 %, respectivamente, lo que la ha situado como la prueba de referencia ^10^.

**Figura 1:**
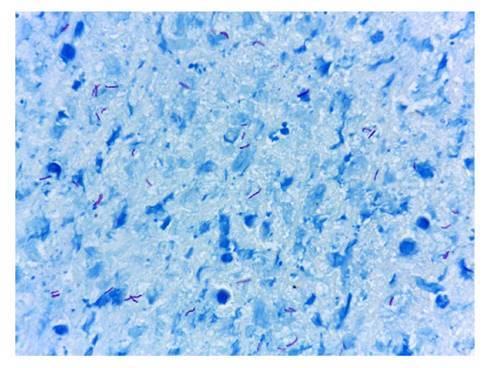
Lavado broncoalveolar, coloración de Ziehl-Neelsen, 100X

Sin embargo, en el caso de la detección de micobacterias en muestras de lavado broncoalveolar, es difícil decidir cuál sería la prueba de referencia: la PCR o el cultivo, lo que puede ser reflejo de la variabilidad de las técnicas en las fases preanalíticas y analíticas. Antes de establecer una de las pruebas como la de referencia, debe considerarse que los resultados de las pruebas no son mutuamente excluyentes en el caso de la detección de micobacterias en el lavado broncoalveolar de pacientes sintomáticos respiratorios, como lo define la Resolución 227 de 2020 del Ministerio de Salud y Protección Social, pues las pruebas son complementarias. Además, la definición operativa de caso de tuberculosis en Colombia establece que la positividad debe verificarse con “alguna” de las pruebas, sin especificar una en particular.

Teniendo esto en cuenta, se compararon las características de la coloración de Ziehl-Neelsen en el Laboratorio de Patología con las de la prueba de PCR y el cultivo en el laboratorio clínico, como métodos diagnósticos en muestras de lavado broncoalveolar.

## Materiales y métodos

### Identificación de casos

**Figura 2:**
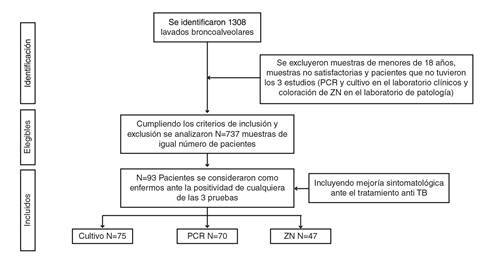
Diagrama de flujo con los criterios de inclusión y exclusión en el estudio y el número de casos detectados, elegibles e incluidos en el análisis

Se tomaron todas las muestras de lavado broncoalveolar enviadas en el 2019 y el 2020 al laboratorio clínico para su cultivo y la identificación del complejo *Mycobacterium tuberculosis* y detección de los genes de resistencia por PCR, las cuales correspondían a 1.308 pacientes (figura 2). Solo se incluyeron las muestras analizadas por PCR y cultivo en el laboratorio clínico y, además, enviadas al Laboratorio de Patología para el análisis citológico y el estudio complementario con coloración de Ziehl-Neelsen.

TB: tuberculosis

Se excluyó a los pacientes menores de 18 años, y a aquellos cuyas muestras no eran satisfactorias o no se habían sometido a las tres pruebas diagnósticas en un mismo procedimiento; finalmente, se analizaron las muestras de 737 pacientes después de cumplir con los criterios de inclusión y exclusión.

La proporción de las características clínicas de los pacientes se comparó utilizando la prueba de ji al cuadrado de Pearson, con un intervalo de confianza del 0,95, considerando como significativo un valor de p<0,05. Las pruebas diagnósticas se compararon en términos de sensibilidad, especificidad y razones de verosimilitud, con un intervalo de confianza de 0,95. Además, se calculó la sensibilidad de las pruebas utilizadas en serie y en paralelo. Todos estos análisis se llevaron a cabo utilizando el *software* R, versión 4.0.3 (2020-10-10) con el paquete DTComPair.

### Coloración de Ziehl-Neelsen

En el Laboratorio de Patología la coloración de Ziehl-Neelsen se hizo según los protocolos de coloraciones especiales del Laboratorio de Patología de la Universidad de Antioquia.

Independientemente de su cantidad, las muestras de lavado broncoalveolar se llevaron a la citocentrífuga (Cytospin™) a 600 rpm durante 5 minutos, hasta obtener los extendidos para la coloración de rutina y las especiales. Para la coloración de Ziehl-Neelsen, las láminas se colocaron sobre una rejilla y el botón de células se cubrió con papel filtro después de la aplicación del carbón fucsina. Posteriormente, las láminas se flamearon hasta lograr la emanación de vapores y se lavaron con agua corriente; luego, se les aplicó alcohol ácido al 1 % hasta obtener un color rosado pálido, se lavaron nuevamente con agua corriente y se les aplicó azul de metileno al 1 % durante un minuto. Las placas se lavaron por última vez con agua corriente antes del montaje de la laminilla cubreobjeto.

### Reacción en cadena de la polimerasa para micobacterias

Antes del tratamiento de las muestras clínicas, se extrajo el material genético y se preparó manualmente la mezcla maestra para la PCR múltiple, siguiendo las instrucciones del fabricante. Los pasos de amplificación y generación de curvas de fusión se hicieron en tiempo real con el sistema PCR CFX-96^TM^ (Bio-Rad) en el instrumento Anyplex II MTB/MDR/XDR^TM^ (Seegene, Corea, Seúl), que detecta *M. tuberculosis* (IS6110 y MPT64) y la resistencia a izoniacida (katG, región promotora del gen *inhA*), rifampicina (rpoB), fluorquinolonas (gryA) y aminoglucósidos (regiones promotoras de los genes *rrs* y *eis*).

Los análisis de los datos de la curva de fusión se hicieron automáticamente y se interpretaron con el programa de visualización Seegene, versión 2.0 (Seegene Technologies, Estados Unidos), utilizando los valores de umbral y de corte predefinidos. En las reacciones se incluyeron controles internos de amplificación positivos y negativos. El tiempo de respuesta de la detección fue de aproximadamente cuatro horas.

### Cultivo para micobacterias

Cada muestra se cultivó en medio líquido y en medio sólido. Las muestras se concentraron por centrifugación durante 30 minutos, se procesaron con el método estándar de N-acetil-L-cisteína e hidróxido de sodio al 2 % (NALC-NaOH) durante 15 minutos a temperatura ambiente ^11^, seguido de centrifugación por 15 minutos a 3.000*g* y resuspensión en solución tampón de fosfato con pH 6,8; el cultivo líquido MGIT se inoculó con 500 μl de muestra y 800 μΙ de PANTA+ OADC BD™ (polimixina B, anfotericina B, ácido nalidíxico, trimetoprim y azlocilina), y se incubó durante 56 días en el equipo BACTEC MGIT 960™, según las instrucciones del fabricante (BD Diagnostics, Sparks, MD, USA). El cultivo sólido se hizo en medio de Lowenstein-Jensen (BBD, Becton-Dickinson) agregando 200 μl de muestra e incubando durante ocho semanas en posición horizontal.

### Consideraciones éticas

Este fue un estudio cuantitativo, no experimental, descriptivo, retrospectivo aprobado por el comité de ética institucional del Hospital San Vicente Fundación. Todos los pacientes incluidos en el estudio residen en Colombia y son mayores de edad.

## Resultados

De las muestras de los 737 pacientes que cumplieron con los criterios de inclusión, 93 fueron positivas, por lo menos, en una de las tres pruebas (Ziehl-Neelsen en el Laboratorio de Patología, PCR o cultivo en el laboratorio clínico), y los pacientes mejoraron con el tratamiento, pues desapareció la sintomatología motivo de consulta y los datos bacteriológicos se ajustaban a lo contemplado en el anexo 3 de la Resolución 227 de 2020 del Ministerio de Salud y Protección Social.

Al integrar los datos de laboratorio y la reacción clínica al tratamiento, la positividad de los pacientes del estudio fue de 12,6 %. De los 93 pacientes con, por lo menos, una prueba positiva (cuadro 1), 43 (46,2 %) eran mujeres y 50 (53,8 %) eran hombres. La edad media de las mujeres fue de 45 años y la de los hombres de 46,3 años. Dieciocho (19,3 %), con una edad promedio de 37,5 años, eran positivos para HIV. De los pacientes positivos para HIV, 16 eran hombres. La diferencia en la proporción de hombres y mujeres positivos para HIV fue estadísticamente significativa (p<0,05).

**Cuadro 1:** Características de los pacientes catalogados como enfermos y de las pruebas diagnósticas (N=93)

		Hombres	Mujeres	p
N=93		50 (53,8 %)	43 (46,2 %)	0,30
Edad promedio		46,3	45	0,736
HIV				*0,0008
	HIV positivo (n=18)	16	2	
	HIV negativo (n=75)	34	41	
Cultivo				0,86
	Cultivo positivo (n=75)	40	35	
	Cultivo negativo (n=18)	10	8	
PCR				0,86
	PCR positiva (n=70)	38	32	
	PCR negativa (n=23)	12	11	
ZN				0,59
	ZN positivo (n=47)	24	23	
	ZN negativo (n=46)	26	20	

PCR: reacción en cadena de la polimerasa para micobacterias

ZN: coloración de Ziehl-Neelsen

En cuanto a la positividad de las pruebas, las tres analizadas fueron positivas en 39 de 93 pacientes. El cultivo fue positivo en 75 de los 93 (80,6 %) pacientes enfermos, con una positividad general de 10,1 % (75/737). En los pacientes positivos para HIV, 17 de 18 (94,4 %) tuvieron cultivos positivos. En el caso de la PCR, esta fue positiva en 70 de los 93 (75,2 %) pacientes enfermos, para una positividad global de 9,5 % (70/737); en cuanto a la relación con los pacientes positivos para HIV, 15 de 18 (83,3 %) fueron positivos en esta prueba. La coloración de Ziehl-Neelsen en el Laboratorio de Patología fue positiva en 47 de los 93 (50,5 %) pacientes enfermos, para una positividad global de 6,37 % (47/737), en tanto que en la población de pacientes positivos para HIV fue de 66,6 % (12/18). Solo se presentó el caso de un paciente de 23 años negativo para HIV, cuyos resultados en el cultivo y la PCR fueron negativos, pero positivos en la coloración de Ziehl-Neelsen en el laboratorio de patología.

El tiempo de entrega de los resultados fue variable. Solo para 5 de los 75 (6,7 %) pacientes con cultivos positivos se obtuvieron los resultados en las primeras cuatro semanas y, para el resto, a las ocho semanas. En cuanto al tiempo de entrega de la PCR para micobacterias, el máximo fue de cuatro horas; este tiempo se mide como un indicador de cumplimiento del laboratorio clínico y, usualmente, el cumplimiento es de 99,9 %. En cuanto la coloración de Ziehl-Neelsen, dada la dinámica de trabajo del laboratorio de patología, donde usualmente no se hacen coloraciones especiales en fines de semana ni festivos, el tiempo promedio de entrega del resultado fue de 3,3 días.

En los enfermos detectados en conjunto por las tres pruebas analizadas (93) (cuadro 2), la comparación de los resultados del cultivo y el total de enfermos arrojó una sensibilidad del cultivo de 0,80, una especificidad de 1,0, una razón de verosimilitud positiva de 1,0 y una negativa de 0,19. En el caso de la PCR, la sensibilidad de la prueba fue de 0,76, la especificidad de 1,0, la razón de verosimilitud positiva de 1,0 y la negativa de 0,24. En la coloración de Ziehl-Neelsen, la sensibilidad de la coloración fue de 0,51, la especificidad de 1,0, la razón de verosimilitud positiva de 1,0 y la negativa de 0,49.

**Cuadro 2:** Resultados de las distintas pruebas diagnóstica en los pacientes enfermos (N=93)

		Enfermos		
		(+)	(-)	Total
Cultivo	positivo	75	0	75
	negativo	18	644	662
		93	644	737
Zn	positivo	47	0	47
	negativo	46	644	690
		93	644	737
PCR	positivo	70	0	70
	negativo	23	644	667
		93	644	737

PCR: reacción en cadena de la polimerasa para micobacterias

ZN: coloración de Ziehl-Neelsen

Al contrastar la positividad de los resultados (cuadro 3) del cultivo y la PCR, de los 75 (de 93) resultados positivos obtenidos en el cultivo, 53 lo fueron también en la PCR; 22 de los positivos en el cultivo fueron negativos en la PCR, y de los 17 negativos en los cultivos, el mismo número fue positivo en la PCR, en tanto que uno fue negativo en ambas pruebas.

**Cuadro 3:** Comparación de resultados positivos entre las pruebas

		PCR		
		(+)	(-)	Total
Cultivo	positivo	53	22	75
	negativo	17	1	18
		ZN		
PCR	positivo	41	29	70
	negativo	6	17	23
		ZN		
Cultivo	positivo	44	31	75
	negativo	3	15	18

PCR: reacción en cadena de la polimerasa para micobacterias

ZN: coloración de Ziehl-Neelsen

Al comparar el cultivo con la coloración de Ziehl-Neelsen, de los 75 (de 93) resultados positivos obtenidos en el cultivo, 44 fueron también positivos en la coloración de Ziehl-Neelsen, en tanto que 31 fueron negativos y, entre los resultados negativos en el cultivo, 3 fueron positivos en la coloración de Ziehl-Neelsen y 15 fueron negativos en ambas pruebas.

Cuando se analizaron los resultados positivos de la PCR (70/93), 41 fueron positivos en la coloración de Ziehl-Neelsen y 29 fueron negativos; de los resultados negativos, 6 fueron positivos en la coloración de Ziehl-Neelsen y 17 fueron negativos en ambas pruebas.

La utilidad de las pruebas al emplearse en serie y en paralelo, se analizó con base en la sensibilidad de las tres combinadas. La sensibilidad en serie y en paralelo del cultivo y de la PCR, fue de 0,60 y 0,95, respectivamente; la del cultivo y la coloración de Ziehl-Neelsen, fue de 0,40 en serie y de 0,90 en paralelo, en tanto que la de la PCR y la coloración de Ziehl-Neelsen, fue de 0,38 en serie y de 0,88 en paralelo. Al comparar los valores de sensibilidad de los cultivos en paralelo y la mejora de la sensibilidad en las diferentes combinaciones (cuadro 4), se obtuvieron valores estadísticamente significativos para la combinación del cultivo y la PCR y de la PCR y la coloración de Ziehl-Neelsen.

**Cuadro 4:** Comparación de sensibilidad de las pruebas

	Serie	Paralelo	Sensibilidad Cultivo	Sensibilidad PCR	Sensibilidad ZN	p
PCR - Cultivo	0,60	*0,95	*0,80	0,76	NA	0,037
ZN - Cultivo	0,40	*0,90	*0,80	NA	0,51	0,061
PCR - ZN	0,38	*0,88	NA	*0,76	0,51	0,022

PCR: reacción en cadena de la polimerasa para micobacterias

ZN: coloración de Ziehl-Neelsen

* Señala los dos valores comparados para la obtención del valor de p.

## Discusión

La positividad de las distintas pruebas diagnósticas para la detección de micobacterias asume diferentes características dependiendo del tipo de muestra. Usualmente, el cultivo se considera la prueba de referencia para el diagnóstico de tuberculosis, y, efectivamente, en nuestro estudio fue la prueba con mayor positividad (75/93) y sensibilidad (0,80). Sin embargo, 17 pacientes con cultivos negativos fueron positivos en la PCR y un paciente fue negativo en el cultivo y en la PCR, pero positivo en la coloración de Ziehl-Neelsen, lo que resalta la complementariedad de las pruebas hechas en paralelo. Además, la sensibilidad de la PCR y la coloración de Ziehl- Neelsen aumentó al combinar las pruebas, pues la sensibilidad de la PCR sola fue de 0,76 y pasó a 0,88 al emplear las dos pruebas, diferencia esta estadísticamente significativa. La combinación de las dos pruebas sobresale especialmente por el tiempo en que podrían obtenerse los dos resultados y el bajo costo de la coloración de Ziehl-Neelsen, lo que compensaría la muy baja positividad del cultivo en las primeras cuatro semanas (6,5 %).

La sensibilidad general de la prueba para el caso de las muestras de esputo varía entre 20 y 60 % y, en el caso del lavado broncoalveolar, no ha superado el 20 % en algunos estudios ^12^, entendiendo que la positividad global de las pruebas depende de la prevalencia de la enfermedad en el grupo especial de estudio. En análisis recientes de la sensibilidad de las coloraciones para bacilos ácido alcohol resistentes en Egipto, tomando como prueba de referencia el cultivo, la sensibilidad fue de 72,1 % y la especificidad de 83,1 % ^13^.

La positividad global de nuestros resultados se diferencia notablemente de los de un estudio en Pakistán, donde se obtuvo una positividad de 49,7 % con la PCR, de 47,8 % con el cultivo y de 40 % con la coloración de Ziehl- Neelsen a partir de muestras pulmonares; en dicho estudio, la PCR obtuvo una mayor positividad que el cultivo, por lo que se la consideraría como la prueba de referencia ^14^. Específicamente en el lavado broncoalveolar, un estudio en el que se utilizó el procesamiento en un equipo Cytospin™ y una modificación del método de coloración de Ziehl-Neelsen, la sensibilidad de la coloración estuvo entre 16,2 y 37,8 %, muy por debajo de la sensibilidad obtenida en nuestro estudio ^15^.

La variabilidad en los resultados de las pruebas, especialmente en cuanto a la positividad global y la sensibilidad, se puede deber a múltiples factores: algunos previos al análisis relacionados con la conservación y el transporte de las muestras que pueden afectar los resultados de las técnicas de detección, así como la preparación y conservación de los reactivos para la coloración de Ziehl-Neelsen y la concentración de las soluciones, así como factores analíticos como la experiencia profesional del personal que interpreta los extendidos ^16^.

Además, la sensibilidad de la prueba está relacionada con la cantidad de bacilos en la muestra; se ha estimado como umbral necesario para la positividad de la coloración de Ziehl-Neelsen un número entre 5.000 y 10.000 bacilos por mililitro ^17^, umbral que puede verse afectado por la fragmentación de la muestra requerida para poder hacer las tres pruebas usuales en paralelo. Para agravar el problema de la sensibilidad, en los niños y en los pacientes coinfectados con HIV, las concentraciones de bacilos por mililitro (paucibacilares) son menores, por lo que el rendimiento de la coloración de Ziehl-Neelsen se reduce en este tipo de pacientes ^18^^,^^19^.

Se necesitan estudios que comparen muestras de diferentes localizaciones anatómicas y las pruebas diagnósticas locales disponibles para la detección de micobacterias, con el fin de conocer las limitaciones y las bondades de cada una de ellas y de su uso en paralelo.

Como conclusión, puede decirse que el uso en paralelo de la PCR y la coloración de Ziehl-Neelsen, mejora de una manera estadísticamente significativa la sensibilidad en la detección de micobacterias en muestras de lavado broncoalveolar, y además, que es una estrategia aplicable debido a la rápida obtención de los resultados de la PCR y el bajo costo de la coloración de Ziehl-Neelsen, comparados con el cultivo.
